# The use of throat packs in pediatric cleft lip/palate surgery: a retrospective study

**DOI:** 10.1007/s00784-018-2387-0

**Published:** 2018-02-22

**Authors:** B. J. A. Smarius, C. H. A. L. Guillaume, G. Jonker, A. B. Mink van der Molen, C. C. Breugem

**Affiliations:** 1 Department of Pediatric Plastic Surgery, Wilhelmina Children’s Hospital, University Medical Center Utrecht, Utrecht, the Netherlands; 2 Division of Pediatric Plastic Surgery, Wilhelmina Children’s Hospital, University Medical Center Utrecht, Heidelberglaan 100, P.O. BOX 85500, 3508 GA Utrecht, the Netherlands; 3 Department of Anesthesia, Wilhelmina Children’s Hospital, University Medical Center Utrecht, Utrecht, the Netherlands; 4 Department of Plastic Surgery, St. Antonius Hospital, Nieuwegein, the Netherlands; 5 Department of Plastic Surgery, Meander Medical Center, Amersfoort, the Netherlands

**Keywords:** Pharyngeal packing, Throat pack, Complications, Cleft lip/palate surgery

## Abstract

**Objectives:**

Throat packs are commonly used to prevent ingestion or aspiration of blood and other debris during cleft lip/palate surgery. However, dislodgement or (partial) retainment after extubation could have serious consequences. The aim of the present study was to investigate the effect of omitting pharyngeal packing during cleft lip/palate surgery on the incidence of early postoperative complications in children.

**Materials and methods:**

A retrospective study was performed on all children who underwent cleft lip/palate surgery at the Wilhelmina Children’s Hospital. This study compared the period January 2010 through December 2012 when pharyngeal packing was applied according to local protocol (group A) with the period January 2013 till December 2015 when pharyngeal packing was no longer applied after removal from the protocol (group B). Data were collected for sex, age at operation, cleft lip/palate type, type of repair, lateral incisions, length of hospital stay, and complications in the first 6 weeks after surgery. Early complications included wound dehiscence, postoperative bleeding, infection, fever, upper respiratory tract infection (URTI), and lower respiratory tract infection (LRTI).

**Results:**

This study included 489 cleft lip/palate operations (group A *n* = 246, group B *n* = 243). A total of 39 (15.9%) early complications were recorded in group A and a total of 40 (16.5%) in group B. There were no significant differences (*P* = 0.902) in complications between the two groups; however, there was a significant difference (*P* < 0.001) in length of hospital stay between the two groups (group A 3.6 days vs group B 3.2 days).

**Conclusion:**

Omitting routine placement of throat packs in cleft lip/palate surgery was not associated with an increased early postoperative complication rate. Therefore, the traditional, routine placement of a throat pack during cleft lip/palate surgery can be questioned.

**Clinical relevance:**

The traditional, routine placement of a throat pack during cleft lip/palate surgery can be questioned.

## Introduction

Throat packs are commonly used in cleft lip/palate surgery. Historically, pharyngeal packing was thought to prevent ingestion or aspiration of blood and other debris during surgery [1–4]. Ingested blood is a potent emetic, and aspirated blood can cause inflammation; both conditions may result in delayed discharge from the hospital.

There is neither convincing evidence that routine pharyngeal packing reduces complications nor that omitting the throat pack increases complications. Several studies described cases of the throat pack inadvertently being left in after extubation, which led to serious postoperative complications [5–8]. In 2012, a child died in the Netherlands when a retained partial throat pack led to acute airway obstruction after extubation. Subsequently, cleft surgeons in the Wilhelmina Children’s Hospital stopped using throat packs since January 2013.

A recent randomized controlled trial was conducted to investigate the association between the use of throat packs and postoperative PONV (postoperative nausea and vomiting) and treat pain after nasal surgery in adult patients [9]. This randomized controlled trial concludes that throat packs do not lower the risk of postoperative nausea and vomiting and are associated with more throat pain in the immediate recovery period after nasal surgery. This study showed that also the use of throat packs in adults can be questioned.

Another study described the postoperative throat effects of nasopharyngeal packing and oropharyngeal packing in adult patients undergoing nasal surgery [10]. This study concluded that the use of nasopharyngeal packing lead to a reduction of throat pain and as well as the incidence of dysphagia compared to oropharyngeal packing.

The relationship between performing cleft lip/palate surgery without throat pack and early postoperative complications in children has not been studied before. This study investigated the effect of omitting pharyngeal packing during cleft lip/palate surgery on the incidence of early postoperative complications in children.

## Patients and methods

### Clinical data

A retrospective study was performed on all children who underwent cleft lip/palate surgery at the Wilhelmina Children’s Hospital in Utrecht, the Netherlands. Patients with only cleft lip repair were excluded from the study. We compared the period January 2010 through December 2012 when pharyngeal packing was applied according to protocol in our hospital (group A) with the period January 2013 till December 2015 when pharyngeal packing was removed from the protocol (group B). Data were collected for sex, age at operation, cleft lip/palate type, type of repair, lateral incisions, length of hospital stay, and complications. This study protocol was approved by the Medical Ethical Board (number 15-583).

### Cleft classification

The cleft palates were classified according to the Veau classification: Veau I (soft cleft palate), Veau II (hard and soft cleft palate), Veau III (soft and hard palates and unilateral cleft of the primary palate), and Veau IV (soft and hard palates and bilateral clefts of the primary palate).

### Length of hospital stay

Day one was defined as the day of admission and subsequent operation.

### Operation technique

All cleft lip/palate repairs were performed according to the standard treatment protocol at our department and were performed by two plastic surgeons. Children with Veau I or Veau II underwent repairs using the Von Langenbeck technique. Von Langenbeck technique in this study was performed with or without relaxing incisions. In case of Veau III or IV, a simultaneous cleft hard palate closure with vomer flap and repair of cleft lip was performed during the first operation. Subsequently, the rest of the cleft palate was closed during a second intervention using the Von Langenbeck technique. For submucous clefts, either Von Langenbeck or Furlow palatoplasty was performed. In case of surgical correction for velopharyngeal insufficiency (VPI), a modified Honig velopharyngoplasty [11], a cranially based posterior pharyngeal flap or a buccal flap procedure either unilateral [12] or bilateral [13] was carried out. A cleft lip repair was performed according to the Fisher or Mulliken technique.

### Anesthetic technique

In general, patients received no premedication. In the vast majority of patients, inhalation induction of anesthesia was performed with sevoflurane. Orotracheal intubation with a cuffed reinforced tube was facilitated by sufentanil and atracurium. Cuff pressure was checked with a manual manometer to obtain no or minimal air leakage at pressures < 20 cm H_2_O. Anesthesia was maintained with sevoflurane and sufentanil in an oxygen/air-mixture with positive pressure ventilation in a circle system. Intraoperatively, intravenous paracetamol, diclofenac, and morphine were administered. Awake extubation was performed in theater after completion of the surgical procedure.

### Complications

All patients were reviewed during hospitalization and at the out-patient clinic 6 weeks postoperatively. Early complications were defined as being evident in the first 6 weeks after surgery. Postoperative complications were categorized as minor, major, and general. In the minor category were partial wound separation/fistula’s (healing without surgical intervention), postoperative bleeding without surgical intervention, and wound infection. The major category includes postoperative bleeding with surgical intervention and partial/complete wound breakdown/fistula (requiring surgical intervention). General complications include fever of unknown etiology, upper respiratory tract infection (URTI), lower respiratory tract infection (LRTI), and airway obstruction.

### Statistics

Patient characteristics were summarized by descriptive statistics. Univariate analysis was used to determine the presence of associations between variables. Fisher’s exact test was used for associations between categorical variables. The Mann-Whitney *U* test was used when one variable was continuous and the other categorical or both variables were continuous. All statistical analyses were performed using IBM Statistical Package for Social Science (SPSS) version 22 (SPSS Inc., Chicago, IL, USA). All calculated *P* values were considered significant if less than 0.05.

## Results

### Characteristics

This study included 489 patients who underwent cleft lip/palate operations (group A *n* = 246, group B *n* = 243). Sixty-three percent (*n* = 156) of the patients in group A and 53% (*n* = 128) of the patients in group B were boys. Mean age at surgery groups A and B were respectively 37.5 months (range 3–211 months) and 37.8 months (range 3–215 months). There was no significant difference in age (*P* = 0.356) between the two groups. There was a significant difference in number of isolated cleft palate surgeries (*P* = 0.002) between group A and group B. No significant differences in number of other operation types between the two groups were observed. Further patient characteristics of the groups are listed in Table 1.

**Table 1:** Patient characteristics

Characteristic	Group A (*n* = 246) *n* (%)	Group B (*n* = 243) *n* (%)	*P* value
Gender			
Male	156 (63)	128 (53)	–
Female	90 (37)	115 (47)	–
Mean age (months)	37.5 (range 3–211)	37.8 (range 3–215)	0.356^
Original cleft type			
VPI	14 (6)	15 (6)	–
Submucous	34 (14)	45 (19)	–
Veau I	7 (3)	5 (2)	–
Veau II	56 (23)	47 (19)	–
Veau III	80 (33)	87 (36)	–
Veau IV	55 (22)	44 (18)	–
Operation type			
Cleft palate closure	130 (53)	94 (39)	0.002^^
Cleft lip/palate closure	38 (15)	49 (20)	0.194^^
Velopharyngoplasty	70 (28)	96 (40)	0.010^^
Fistula closure	8 (3)	4 (2)	0.382^^

*VPI*, velopharyngeal insufficiency

^Mann-Whitney *U* test

^^Fisher’s exact test

### Early complications

Overall, 39 (15.9%) early complications were registered in group A and a total of 40 (16.5%) in group B. There was no significant difference in overall early complications between the two groups (*P* = 0.902).

There was a minor complication rate of 7.7% (*n* = 19) in group A and 10.7% (*n* = 26) in group B, a major complication rate of 3.3% (*n* = 8) in group A and 3.3% (*n* = 8) in group B, and a general complication rate of 4.9% (*n* = 12) in group A and 2.5% (*n* = 6) in group B. Partial dehiscence without surgical intervention was the most frequent complication (Table 2).

**Table 2:** Minor, major, and general complications

Characteristic	Group A (*n* = 246) *n* (%)	Group B (*n* = 243) *n* (%)	*P* value
Early complications			
No	207 (84.1)	203 (83.5)	
Yes	39 (15.9)	40 (16.5)	0.902^
Minor complications			
Partial wound dehiscence/fistula*	18 (7.3)	22 (9.1)	0.513^
Postoperative bleeding*	0 (0.0)	2 (8.2)	0.246^
Wound infection	1 (0.4)	2 (8.2)	0.622^
Major complications			
Partial/complete wound breakdown or fistula**	8 (3.3)	6 (2.5)	0.787^
Postoperative bleeding**	0 (0.0)	2 (0.8)	0.246^
General complications			
Fever e.c.i.	2 (0.8)	3 (1.2)	0.685^
URTI***	8 (3.3)	3 (1.2)	0.221^
LRTI****	0 (0.0)	0 (0.0)	–
Airway obstruction	2 (0.8)	0 (0.0)	0.499^

*Without surgical intervention/secondary healing

**With surgical intervention

***URTI, upper respiratory tract infection

****LRTI, lower respiratory tract infection

^Fisher’s exact test

Of 39 early complications in group A, 29 (74.4%) occurred after cleft palate closure, 4 (10.3%) after lip/palate (vomer) closure, 3 (7.7%) after velopharyngoplasty, and 3 (7.7%) after fistula repair. Of 40 complications in group B, 26 (65%) arose after cleft palate closure, 4 (10%) after lip/palate closure, 9 (22.3%) after velopharyngoplasty, and 1 (2.5%) after fistula repair. Complication rates and details by operation type are given in Tables 3 and 4.

**Table 3:** Complication rate by operation type

Operation type	Complications group A *n* (%)	Complications group B *n* (%)	*P* value^
Cleft palate surgery			
With throat pack (*n* = 130)	29 (22.3%)	–	
Without throat pack (*n* = 94)	–	26 (27.7%)	0.432
Cleft lip/palate surgery			
With throat pack (*n* = 38)	4 (10.5%)	–	
Without throat pack (*n* = 49)	–	4 (8.2%)	0.725
Velopharyngoplasty			
With throat pack (*n* = 70)	3 (4.3%)	–	
Without throat pack (*n* = 96)	–	9 (9.4%)	0.243
Fistula closure			
With throat pack (*n* = 8)	3 (37.5%)	–	
Without throat pack (*n* = 4)	–	1 (25%)	1.000
Total complications	39	40	

^Fisher’s exact test

**Table 4:** Complications details by operation type

Operation type	Complications group A *n* (%)	Complications group B *n* (%)	*P* value^
Cleft palate surgery			
Partial wound dehiscence/fistula*	12 (30.8)	19 (47.5)	0.198
Wound infection	–	1 (2.5)	0.479
Partial/complete wound breakdown or fistula**	8 (20.5)	4 (10.0)	0.382
URTI***	7 (17.9)	2 (5)	0.176
Airway obstruction	2 (5.1)	–	0.499
Cleft lip/palate surgery			
Partial wound dehiscence/fistula*	2 (5.1)	2 (5)	1.000
Wound infection	1 (2.6)	–	1.000
Partial/complete wound breakdown or fistula**	–	1 (2.5)	0.479
Fever e.c.i.	–	1 (2.5)	0.479
URTI***	1 (2.6)	–	1.000
Velopharyngoplasty			
Partial wound dehiscence/fistula*	1 (2.6)	–	1.000
Postoperative bleeding*	–	2 (5)	0.246
Wound infection	–	1 (2.5)	0.479
Partial/complete wound breakdown or fistula**	–	1 (2.5)	0.479
Postoperative bleeding**	–	2 (5)	0.246
Fever e.c.i.	2 (5.1)	2 (5)	1.000
URTI***	–	1 (2.5)	0.479
Fistula closure			
Partial wound dehiscence/fistula*	3 (7.7)	1 (2.5)	0.623
Total complications	39 (100)	40 (100)	

*Without surgical intervention/secondary healing

**With surgical intervention

***URTI, upper respiratory tract infection

^Fisher’s exact test

In group A, there were two cases of acute airway obstruction. None of the two airway obstructions were directly related to the use of a throat pack. One syndromic child was known with central apneas and was admitted to the pediatric intensive care unit (PICU) for postoperative monitoring. The other child had an airway obstruction after extubation as a result of non-bloody sputum aspiration in the recovery room and was admitted to PICU for monitoring overnight.

The mean length of hospital stay in group A and B was 3.6 days (range 2–32 days, 95% CI [3.27–3.99]) and 3.2 days (range 2–26 days, 95% CI [2.94–3.43]), respectively. This was a statistically significant difference in length of stay between the two groups (*P* < 0.001).

## Discussion

Throat packs are commonly used during cleft lip/palate surgery because of the theoretical advantage of reducing the possibility of aspiration of blood and secretions [1–4]. During oral surgery, non-suctioned blood may flow through the nasopharynx and oropharynx and may drain into the stomach or leak past the endotracheal tube cuff into the airway. This drainage is facilitated by the reverse Trendelenburg position in bed postoperatively. Cuffed tracheal tubes do not provide 100% protection from aspiration [14]. It is assumed that pharyngeal packing will protect from ingestion or aspiration of blood and other debris during surgery [1–4]. However, it has been shown that pharyngeal packing does not offer 100% protection [3]. In addition, the placing of a pharyngeal pack is associated with complaints of postoperative painful throat [15–18] and trauma and edema in oral and pharyngeal structures after surgery [16, 17, 19]. Moreover, the risk of leaving the pack inadvertently in place after extubation can lead to acute airway obstruction [6, 7], intestinal occlusion, as well as complications such as oral aphtosis and acute tongue enlargement [20–22].

Although postoperative nausea and vomiting does not fall within the scope of this study, there is evidence that pharyngeal packing does not reduce the amount of nausea and vomiting [16, 17, 23].

In our study, 246 children underwent cleft lip/palate surgery with pharyngeal packing and 243 children underwent cleft lip/palate surgery without pharyngeal packing. Early postoperative complication rate in our study was 15.9% (with throat pack) and 16.5% (without throat pack). This is comparable to the incidence of early postoperative complications reported in the literature, which varies between 3.9 and 35.8% (Table 5). In none of the studies, the usage of a throat pack is mentioned, but it could be assumed that all patients received a throat pack. Nevertheless, we found no difference in complication rate between the throat pack and no throat pack groups in our study.

**Table 5:** Overview articles early postoperative complications after lip/palate surgery

Article author (date)	Patients/surgeries (*n*)	Cleft type	Use throat pack	Early complication rate (%)
Adesina et al. (2016)	120	UCL, BCL, CP	Not described	35,8%
Schönmeyr et al. (2016)	1408	CP	Not described	16.9%
Park et al. (2016)	338	SMCP, ICP	Not described	18.3%
Schönmeyr et al. (2015)	2062	UCL, BCL	Not described	4.4%
Deshpande et al. (2014)	709	CP	Not described	3.9%
Zang et al. (2014)	2100	CL, CP	Not described	6.5%
Lees and Pigott (1992)	164	CL, CP	Not described	26.2%

*UCL*, unilateral cleft lip; *BCL*, bilateral cleft lip; *CL*, cleft palate; *CP*, cleft palate; *SMCP*, submucous cleft palate; *ICP*, incomplete cleft palate

It is noticeable that our study demonstrated a significant difference in length of hospital stay between the two groups (*P* < 0.001). The mean length of hospital stay in group A and B was respectively 3.6 days (range 2–32 days, 95% CI [3.27–3.99]) and 3.2 days (range 2–26 days, 95% CI [2.94–3.43]). We do not feel this shorter admission of 0.4 day to be of clinical relevance. It is known that patients are nowadays admitted for shorter periods of time when compared to historical control groups. Since our admission and discharge policies have not changed during the study period (2010–2015), we do not feel this will influence our results. Earlier studies demonstrated postoperative sore throat, edema, and small mucosa tears from the throat pack placement [16, 17, 19, 24]. Although not within the scope of this study, these side effects may have influenced the length of hospital stay in group A.

Partial dehiscence after cleft palate repair in Veau I or II using Von Langenbeck technique was the most common complication in this study. Partial dehisence occurred less frequently in Veau III and IV. This difference may be explained by the use of the vomer flap technique in Veau III and IV. Nowadays, the vomer flap is often used for early partial hard palate closure. This regional flap is easily accessible and located next to the cleft palate. It is well vascularized and is useful in the majority of cleft palate patients [25]. It is a suitable and an effective procedure during cleft palate closure [26–28].

There was a significant difference in number of isolated cleft palate surgeries (*P* = 0.002) between group A and group B. We do not feel this difference in number operation type is of clinical relevance for this study.

During the reconstructive surgeries, a cuffed tube was used in all cases. Although cuffed tubes reduce the risk of (micro) aspiration, they may not provide 100% protection from aspiration [14]. It is imperative to suction pharyngeal blood prior to extubation to prevent aspiration.

In our opinion, only when severe blood loss is anticipated (e.g., bleeding disorders), the use of a throat pack is incumbent. If the surgeon and anesthesiologist have decided to use a throat pack in the patient’s best interest, an appropriate risk management strategy should be used to minimize the hazard of retention. Everyone in the team (surgeon, anesthesiologist, nurses) must be informed and a strategy that ensures the removal of the throat pack before extubation agreed upon. To avoid potential error, the packs should be placed and removed by the same person [29].

Al-Lami et al. concluded that the use of throat packs does not confer PONV reduction benefit after nasal surgery in adult [9]. Postoperatively, patients filled out a validated questionnaire measuring degree of PONV and throat pain. The use of throat pack, however, was associated with a small but statistically significant more throat pain. This study showed that also the use of throat packs in adults can be questioned. Our study was focused on children (mean age of 37.5 and 37.8 months). It is hard to conduct a survey in children at this young age to measure the degree of PONV and throat pain.

This study has several limitations. Due to its retrospective nature, there are inherent weaknesses; however, comparisons to the published literature can be made. Its strengths include the large study population, a control group, and the fact that patients were operated by two surgeons with comparable technique, follow-up, and postoperative care.

## Conclusion

This study demonstrates that omitting pharyngeal packing during cleft lip/palate surgery does not lead to an increased early postoperative complication rate when compared to patients in whom a throat pack was used. Therefore, the traditional, routine placement of a throat pack during cleft lip/palate surgery can be questioned.
